# Preventing Self-Harm Among Adolescents Through Culturally Adapted Psychological Intervention in Pakistan: A Multicenter RCT

**DOI:** 10.1192/bjo.2024.165

**Published:** 2024-08-01

**Authors:** Sehrish Tofique, Nasim Chaudhry, Tayyeba Kiran, Nadeem Gire, Nusrat Husain

**Affiliations:** 1 Pakistan Institute of Living and Learning, Karachi, Pakistan; 2 Pakistan Institute of Living and Learning, Rawalpindi, Pakistan; 3 National Center for Research on Suicide Prevention, Rawalpindi, Pakistan; 4 University of Bolton, Manchester, United Kingdom; 5 University of Manchester, Manchester, United Kingdom

## Abstract

**Aims:**

Self-harm is the preventable cause of premature death by suicide. In adolescents suicide is the fourth leading cause of death and Pakistan is one of the youngest nations in the world. Culturally relevant solutions for the prevention of self-harm among adolescent are almost non-existent in LMICs. The aim of this trial is to assess the clinical and cost-effectiveness of a culturally adapted manual assisted problem-solving intervention for youth (YCMAP) with history of self-harm (within 3 months) to reduce self-harm repetition over the period of 12 months.

**Methods:**

This was a rater-blind, multicenter randomised controlled trial, with a nested qualitative component to explore perceived usefulness of the intervention from the perspective of different stakeholders. Primary care centers, emergency departments, medical units from participating healthcare facilities in Karachi, Hyderabad, Lahore, Rawalpindi and Multan, Pakistan served as recruitment sites in addition to self-referrals. Patients with a recent history of self-harm (*n* = 684) were assessed and randomised (1:1) into either of the two trial arms; YCMAP with enhanced treatment as usual (E-TAU) or E-TAU. The YCMAP is a manualized, psychological intervention based on problem-solving therapy, principles of cognitive behavior therapy (CBT), dialectical behavior therapy (DBT), psychoeducation, and a comprehensive assessment of the self-harm attempt using stories of four young people, comprising 8–10 one-to-one sessions delivered over three months by trained therapists. Primary outcome was the reduction in the self-harm repetition at 12-month post-randomisation and secondary outcomes were distress, suicidal ideation, hopelessness, health-related quality of life (QoL), and level of satisfaction with service received, assessed at baseline, 3-, 6-, 9-, and 12-month post-randomisation.

**Results:**

We screened 1099 young people for eligibility and 684 eligible, consented patients were randomly assigned to the YCMAP plus E-TAU arm (*n* = 342) and E-TAU arm (*N* = 342). Improvement in repetition rates of self-harm, hopelessness, suicidal ideation and psychological distress were clinically significant in YCMAP arm as compared with E-TAU. Thematic analysis of interviews with adolescents who participated in the intervention arm (N = 20) highlighted the intervention as useful in improving their mental health and well-being, and easy to understand.

**Conclusion:**

Adolescents are an important target population for the prevention of suicide and other mental health problems. Implementation strategies are needed such as digitalization of culturally adapted manual assisted psychological interventions or task shifting approach for scalable suicide prevention interventions in low resource settings like Pakistan to meet mental health needs.

